# Production of kojic acid by *Aspergillus flavus* OL314748 using box-Behnken statistical design and its antibacterial and anticancer applications using molecular docking technique

**DOI:** 10.1186/s12866-024-03289-2

**Published:** 2024-04-25

**Authors:** Ghada Abd-Elmonsef Mahmoud, Abo bakr Abdel Shakor, Nahla A. Kamal-Eldin, Abdel-Naser A. Zohri

**Affiliations:** 1 Botany and Microbiology Department, Faculty of Science, Assiut University, Assiut, P.O 71516 Egypt; 2 Zoology Department, Faculty of Science, Assiut University, Assiut, P.O 71516 Egypt

**Keywords:** Antibacterial agents, Anticancer agents, Molecular docking, Box-Behnken design, *Aspergillus flavus*, Kojic acid

## Abstract

Kojic acid is a wonderful fungal secondary metabolite that has several applications in the food, medical, and agriculture sectors. Many human diseases become resistant to normal antibiotics and normal treatments. We need to search for alternative treatment sources and understand their mode of action. *Aspergillus flavus* ASU45 (OL314748) was isolated from the caraway rhizosphere as a non-aflatoxin producer and identified genetically using 18S rRNA gene sequencing. After applying the Box-Behnken statistical design to maximize KA production, the production raised from 39.96 to 81.59 g/l utilizing (g/l) glucose 150, yeast extract 5, KH_2_PO_4_ 1, MgSO_4_.7H_2_O 2, and medium pH 3 with a coefficient (*R*^*2*^) of 98.45%. Extracted KA was characterized using FTIR, XRD, and a scanning electron microscope. Crystalized KA was an effective antibacterial agent against six human pathogenic bacteria (*Bacillus cereus, Staphylococcus aureus, Escherichia coli, Klebsiella pneumonia, Serratia marcescens,* and *Serratia plymuthica*). KA achieves high inhibition activity against *Bacillus cereus, K. pneumonia,* and *S. plymuthica* at 100 μg/ml concentration by 2.75, 2.85, and 2.85 compared with chloramphenicol which gives inhibition zones 1, 1.1, and 1.6, respectively*.* Crystalized KA had anticancer activity versus three types of cancer cell lines (Mcf-7, HepG2, and Huh7) and demonstrated high cytotoxic capabilities on HepG-2 cells that propose strong antitumor potent of KA versus hepatocellular carcinoma. The antibacterial and anticancer modes of action were illustrated using the molecular docking technique. Crystalized kojic acid from a biological source represented a promising microbial metabolite that could be utilized as an alternative antibacterial and anticancer agent effectively.

**Supplementary Information:**

The online version contains supplementary material available at 10.1186/s12866-024-03289-2.

## Introduction

Kojic acid acts as an organic acid and is created as a secondary biological metabolite during the aerobic fermentation of different foods by various types of fungi. Around 58 different fungus strains have been employed to produce kojic acid, particularly those belonging to *Aspergillus, Penicillium*, *Mucor,* etc. [1]. The chemical identification of KA is 5-hydroxy-2-hydroxymethyl-ɣ-pyrone [2]. The molecular formula is C_6_H_6_O_4_. It is a flammable white crystalline odorless powder with a molecular weight of 141.1 [3] and a melting point of 151 °C–154 °C [4]. KA is soluble in ethanol, water, and ethyl acetate; however, it has less solubility through chloroform, ether, and pyridine [5]. It is interactive at almost all the ring positions, and generates significant industrial chemical products like azo dyes, metal chelates, pyridines, and ethers [3, 5]. It has weak acidic capabilities that enable it to combine with several metal ions forming salts [6], and its maximum UV absorption was demonstrated at 280–284 nm [7]. It has crystalized properties that result in needle structures [8].

Kojic acid is fundamentally produced by *Aspergillus flavus* [9–13], *A. flavus* var. *columnaris* [11, 14], *A. oryzae* [15–17], *A. oryzae* var*. effusus* [18], *A. tamarii* [14, 19], and *A. parasiticus* [9, 14, 20]. It was utilized as an anti-browning agent, particularly in soy sauce, miso, sake preparations [21, 22], and for the preservation of canned foods to prevent browning, and as a flavor enhancer [23, 24]. KA inhibits the melanin build in human skin through inhibition of tyrosinase enzyme activity which was in charge of melanin synthesis [25, 26]. KA was used in skin care products as de-pigmenting and skin-lightening products like gels, creams, sunscreens, bath salts, and baby lotions [27, 28]. Moreover, the combination of KA with chitosan was utilized in biodegradable plastics industries [29, 30], and the combination with vanadium utilized it as an anti-diabetic agent [31].

Infectious illnesses especially by bacteria have become more complex and frequently hard to cure, which expands the morbidity and even mortality [32]. Although, antibiotics are utilized for both the avoidance and curing of bacterial diseases, uncontrolled use generates antibiotic bacterial resistance [33]. In a situation when microbes resist the medicine the speeding of infection spread makes infections challenging to treat [34]. The resistance rates of *Klebsiella pneumoniae, Escherichia coli,* and *Staphylococcus aureus* are increasing every day from 4% reaching 92.9% resistance to usually utilized antibiotics [32, 35]. Despite these issues, no new antibiotics were discovered during the last few years [32], and researchers need to seek for new antimicrobial agents to overcome the bacterial resistance issue. Kojic acid has recently attracted interest due to its antimicrobial and insecticidal qualities [36]. Kojic acid acts as an effective antimicrobial agent against many fungi and bacteria comparatively than antibiotics like ceftazidime and nitrofurantoin, because of its acidic nature [20, 37]. It could inhibit several bacterial genera like *Aerobacter, Corynebacterium, Bacillus, Clostridium, Diplococcus, Micrococcus, Klebsiella, Escherichia, Neisseria, Pseudomonas, Chromobacterium, Staphylococcus, Proteus,* and *Vibrio* [38]. Kojic acid was utilized as an active Tubercle bacilli inhibitor in humans [39]. Previous studies had shown that KA was more active in Gram-negative bacteria than positive ones [40, 41].

Kojic acid can be taken against aging and cancer as an oral medicine; KA from *A. tamarii* MM11 was demonstrated as an effective antioxidant agent with high cytotoxic activity to human liver cancer (HepG-2 cell line), confirming their strong antitumor activity to hepatocellular carcinoma [42]. Another study indicated that KA combination therapy with Mannich base (ciprofloxacin) has a significant antitumor activity to HepG-2 [43]. It has a suppressive effect on cancerous cell proliferation [44]. Halogenated KA derivatives were utilized also in leukemia treatment [45]. It was revealed that 5-OH-2Chloromethyl-4-pyran-4-one and 5-benzyloxy-2-thriocyanatomethyl-4-pyran-4-one inhibits the genetic materials, cytoplasmic phosphorylation, and protein synthesis that reduces the growth of neoplastic cells [46, 47]. Seven kojic acid derivatives were cytotoxic in HepG-2 cell line [48]. Human skin (A431) and human breast (MCF7) carcinoma cells were treated earlier with derivatives of KA like 5-benzyloxy-2-selenocyanatomethyl4-pyranone (P763) and 5-methoxy-2-selenocyanatomethyl-4-pyranone (P764) for 24, 48 and 72 h by Fickova et al. [49] and he stated that they cause intracellular huge injury to mitochondria and lysosomes with the highest growth inhibitory by P763 in both cell lines. Azidometalkojates zinc derivatives exhibit cytotoxic activity against human HeLa tumor cells [3].

Previous studies focused on the antibacterial effect of chemically synthesized KA, while the anticancer activity of biological KA wasn’t investigated in most previous researches only a few tested the activity of crude KA on HepG-2. The present work aims first to enhance KA production using statistical experimental design, extract, and crystallized KA from *A. flavus* ASU45 (non-aflatoxin producer). Second, investigate the antibacterial and anticancer activities of crystalized KA (from *Aspergillus flavus* ASU45) against six human pathogenic bacteria and three types of cancer cell lines; mammary carcinoma (Mcf-7), and hepatocellular carcinoma (HepG2, and Huh7) comparison with chemically synthesized KA. Third clear the possible antibacterial and anticancer mechanisms of KA using the molecular docking technique as a bioinformatics tool.

## Materials and methods

### *Aspergillus*, aflatoxins testing and identification

Fifty-two rhizospheric fungal isolates were tested for their KA production capabilities [14]. Six isolates were highly kojic acid producers and were tested for aflatoxins production according to El-Kady and Moubasher [50]. Sterilized potato glucose broth medium (20 g potato and 2 g glucose in 100 ml distilled water) inoculated with 2% inoculum (2 × 10^5^ spore/ml) and incubated at 28 ± 1 °C. After 10 days, the cultures were mixed with the same volumes of chloroform for 10 min, filtrated, then the chloroform layer separated and evaporated to 1 ml. Thin layer chromatography (TLC) was used for aflatoxins detection on a silica gel plate (60 GF254) injected with extracts, and put in a glass chamber containing chloroform: acetone (90:10, v:v). The plate then was examined at 254 and 356 nm [17]. *Aspergillus flavus* ASU45 was molecularly identified using the universal primers ITS1 and ITS4 in SolGent, Daejeon, Korea following White et al. [51] method. Sequences were analyzed by BLAST via the National Center of Biotechnology Information (NCBI) and compared to sequences from closely related species before being placed in the GenBank database with a unique accession number [52].

### Optimizing kojic acid production using box-Behnken statistical design

Modified Czapek’s glucose broth medium was used as fermentation medium (g/100 ml): glucose 10; KH_2_PO_4_, 0.1; yeast extract, 0.5; and MgSO_4_, 0.05, dissolved in 100 ml distilled water with initial pH 3 [17]. Box–Behnken experimental design was assessed for enhancing the production by *A. flavus* ASU45 via studying the interactions between different parameters, and the main and quadratic effects of tested variables. Five parameters, 3-levels randomized statistical design with 41 runs were conducted exploring. Glucose (100, 150, 200; A), yeast extract (1, 5, 10; B), KH_2_PO_4_ (0, 1, 3; C), MgSO_4_ ·7H_2_O (0, 0.5, 2; D) g/l and pH (3, 5, 7; E) were stated by three levels low, medium, and high concentrations (− 1, o, + 1). The non-linear quadratic design was assessed by the coming quadratic Eq. (1) following Yan et al. [53].

1 $$Y={\upbeta}_0+\sum {\upbeta}_{\textrm{i}}\ {\textrm{x}}_{\textrm{i}}+\sum {\upbeta}_{\textrm{ii}}\ {{\textrm{x}}_{\textrm{i}}}^2+\sum {\upbeta}_{\textrm{ij}}{\textrm{x}}_{\textrm{ij}}$$

Y; predicted KA values, β_0_; intercept, β_i_; linear impact, β_ii_; squared impact, β_ij_; interaction impact, and x_i,_ x_ij_; independent variables levels. The relationships between the variables were made clear using response surface plots, curves of actual and expected values, and statistical analysis of all data. Derringer’s necessary equations were established to ensure the accuracy of the developed models.

### Extraction, crystallization and characterization of crystalized kojic acid

Ten days cultures of *A. flavus* ASU45 growing on the optimum modified Czapek’s glucose broth medium were filtrated, centrifuged at 5000 xg for 10 min., and extracted using ethyl acetate (1:1, filtrate: solvent). KA was measured according to Sanjotha et al. [13] by ferric chloride reagent at 540 nm using a spectrophotometer. For crystallizing KA; ethyl acetate extracts were stored for 1 day at 5 °C, then evaporated using a rotatory evaporator at 70 °C (120 rpm), and then KA crystals were removed in clean filter paper until complete drying [52, 54]. Kojic acid crystals were characterized using FTIR, XRD, and scanning electron microscope. IR spectral data were revealed in spectrophotometer type Nicolet iS10 (4000–500 cm^−1^), PXRD pattern was revealed in the 2θ range of 10–80° via Philips PW 1710 X-ray diffractometer working at 40 kV and 40 mA (λ = 1.54060A°) equipped with nickel filtered CuKα radiation. Kojic acid crystals were photographed by scanning electron microscopy (SEM) (JSM 5400 LV; JEOL, Japan).

### Antibacterial activity of crystallized kojic acid

Antibacterial activities of crystalized KA (from *Aspergillus flavus* ASU45) were tested on six different human pathogenic bacteria with concentrations 0, 25, 50, 75, and 100 μg/ml KA compared with standard KA (chemically synthesized) using the well-diffusion method following Saleh et al. [39]. The tested bacterial isolates were 2 Gram +ve bacteria (*Bacillus cereus* ASU 300*,* and *Staphylococcus aureus* ASU 301) and 4 Gram -ve bacteria (*Escherichia coli* ASU302*, Klebsiella pneumonia* ASU 303*, Serratia marcescens* ASU 304*,* and *Serratia plymuthica* ASU 305). The isolates were cultivated, individually, in a nutrient broth medium and incubated for 24 hours at 30 °C ± 1 [55, 56]. One ml of each bacterial suspension (1 × 10^6^ CFU/ml) was spread on nutrient agar medium, then, using sterilized cork polar (0.5 cm), 3 wells were created on the medium of each Petri dish and filled with 50 μl of crystalized or standard KA dissolved in ethyl acetate. Chloramphenicol was utilized as a positive control with 100 μg/ml concentration and ethyl acetate served as a negative control. After 48 hours of incubation at 30 °C ± 1, the growth inhibition was recorded as a clear zone around the wells.

### Anticancer activity of crystallized KA

Anticancer activities of crystalized KA compared with standard KA (0, 25, 50, and 100 μg/ml) were tested in Mcf7, HepG2, Huh7 cell lines. DMEM medium containing L-glutamine, fetal bovine serum, and 10.000 units of penicillin/ml and streptomycin as antibacterial was obtained from Gibco (Invitrogen, CA. USA). Ethidium bromide (EB) and acridine orange (AO) were purchased from Sigma (St. Louis, MO, USA). Cell lines were purchased from VACSERA – Cell Culture Unit (Dokky, Giza, Egypt), which was originally collected from an American Type Culture Collection (ATCC). All the experiments were carried out using cells free from mycoplasma. The cells were cultured in DMEM medium containing L-glutamine supplemented with 10% (v/v) heat-inactivated FBS, 100 μg/ml streptomycin, and penicillin at 37 °C in 95% air and 5% CO_2_.

#### MTT assay

Mcf7, HepG2, Huh7 cells (2 × 10^3^ cells/well) were incubated for 24 h. with 0, 25, 50, and 100 μg/ml crystallized and standard KA concentrations in 96-well plates. The medium was substituted with fresh medium added to it 2 mg/ml MTT and incubated for 3 h. at 37 °C in 200 μl of DMSO, until formazan crystals dissolved. The quantification was performed by measuring the absorbance at 570 nm. Data were expressed compared with the control as the average percentage of viable cells [57].

The percentage of cell viability was calculated following Eq. (2):

2 $$\%\textrm{Viability}=\left(\left(\textrm{Mean}\ \textrm{OD}\ \textrm{sample}\right)/\left(\textrm{Mean}\ \textrm{OD}\ \textrm{blank}\right)\right)\times 100$$

#### Acridine Orange/ethidium bromide staining assay

HepG2 cells were plated on a 6-well plate (0.5 × 10^6^ cells/ml), after 24 hours, the medium was aspirated and fresh medium containing crystallized and standard kA (0, 50, 100 μg/ml) was added and incubated for 24 hours. Then cells were stained for 5 minutes with 1% AO/EB dye, washed trice using 1X PBS, and examined under a fluorescence microscope.

#### Scratch assay

A culture of HepG2 cells was loaded in 6-well plates with 5 × 10^5^ cells/well density and cultured in DMEM, at 37 °C for 24 hours. After aspirating the culture medium, the confluent monolayer of cells was scratched using a sterile pipette tip. A fresh medium containing crystallized or standard KA (0, 50 μg/ml) was poured over the cells and cultured for another 24 hours. Phase contrast images of the scratch wound were taken at 0 and 24 hours after treatment by KA using a phase contrast microscope. Using Image J software, image analysis was performed to calculate the wound closure rate.

### Molecular docking of KA as antibacterial and anticancer agent

Preparation of targeted proteins and ligands; the binding site of tyrosinase crystal structure and nuclear factor kappa B (NFK_b_) crystal structure were assessed from the co-crystallized ligand, through crystal protein (PDB codes: 6EI4, found at https://www.rcsb.org) by using MOE 19.0901 Software. First, the molecules of water were removed from the protein complex, and the protein energy was minimized via the application of MMFF94 force fields. Then the binding site rigid structure was acquired through fixed atom constraint implementation. Finally, the essential amino acids of the target protein were defined and processed for docking. Tested ligands 2D structures were figured via Chem-Bio Draw Ultra17.0, USA, and conservative in MDL-SD format (MOE 19.0901 Software, USA). 3D structures were generated, charged fixed and the force field (MMFF94) 0.05 RMSD kcal/mol. Was applied to minimize the energy.

Molecular docking and validation: the protein receptor was formed, and the prepared ligands were chosen for the molecular docking process, the placement, and refinement, were scored through London dG and GBVI/WSA dG. The receptor was set rigid however ligands were left flexible. In the refinement each molecule establishes 10 protein interactions, then docking based on the interaction energy selected best-fitted poses with tyrosinase crystal structure and nuclear factor kappa B active sites and the 3D draw was assessed. All previous processes were stetted to predict the binding mode, affinity, orientation of docking pose and the free energy (∆G) binding of tested compounds. For molecular docking validation; re-docking of co-crystallized ligand by the active site of the receptor, calculation of the root mean square deviation (RMSD) was conducted for the reliability and the reproducibility of the docking algorithm. Co-crystallized ligands were redocked in targeted sites, B5N (crystal ligand) redocked in crystal structure of tyrosinase (6EI4) with RMSD value 1.22 Å, and crystal structure of NFK_b_ *(*target site PDB ID: 5T8P) with RMSD value 0.89 Å.

### Statistical analysis

The Box–Behnken statistical design data were analyzed utilizing American Design Expert 7.0.0 statistical software, data were analyzed via quadratic regression and one-way ANOVA with a *P* value of 0.05 for variable interaction analysis. Also, the antimicrobial data was analyzed using one-way ANOVA using Statistix 8.1 software with a *P* value of 0.05.

## Results

### *Aspergillus*, aflatoxins testing and identification

Among six *Aspergillus flavus* group isolates, only two isolates (*Aspergillus flavus* ASU45 and *Aspergillus oryzae* ASU44) couldn’t produce aflatoxin. *Aspergillus flavus* ASU45 isolated from caraway was selected depending on KA production (39.96 g/l KA) and identified genetically using the universal primers ITS1 and ITS4**.** ITS sequencing of *Aspergillus flavus* showed high similarity (100%) with GenBank accession numbers, *Aspergillus flavus* (Accession no. MT645322.1), *A. flavus*, (Accession no. MT447477.1), *A. flavus* (CP051065.1), and *A. flavus* (MN547373.1) and was identified as *Aspergillus flavus* ASU45 (Accession no. OL314748).

### Optimizing kojic acid production using box-Behnken statistical design

Kojic acid production by *A. flavus* ASU45 (OL314748) in modified Czapek’s glucose broth medium was optimized and evaluated using a Box–Behnken statistical experimental design. Five fermentation medium parameters involving glucose (100, 150, 200; A), yeast extract (1, 5, 10; B), KH_2_PO_4_ (0, 1, 3; C), MgSO_4_ ·7H_2_O (0, 0.5, 2; D) g/l and pH (3, 5, 7; E), with three-level were tested by 41-runs (Table S1). KA predicted values were calculated via Eq. (2) for second-order polynomial:

3 $$\textbf{Kojic}\ \textbf{acid}\ \left(\textbf{g}/\textbf{l}\right)=55.8+(6.54)\ \textrm{A}+(4.76)\ \textrm{B}+\left(-0.44\right)\ \textrm{C}+\left(-0.58\right)\ \textrm{D}+\left(-21.08\right)\ \textrm{E}+(9.17)\ \textrm{AB}+\left(-3.29\right)\ \textrm{AC}+(2.13)\ \textrm{AD}+(1.03)\ \textrm{AE}+\left(-5.66\right)\ \textrm{BC}+\left(-5\right)\ \textrm{BD}+(0.54)\ \textrm{BE}+\left(-0.24\right)\ \textrm{CD}+1.53\Big)\ \textrm{CE}+(0.91)\ \textrm{DE}+\left(-6.5\right)\ \textrm{A}^2+\left(-8.52\right)\ \textrm{B}^2+\left(-4.79\right)\ \textrm{C}^2+\left(-5.09\right)\ \textrm{D}^2+(8.96)\ \textrm{E}^2$$

**Fig. 1:**
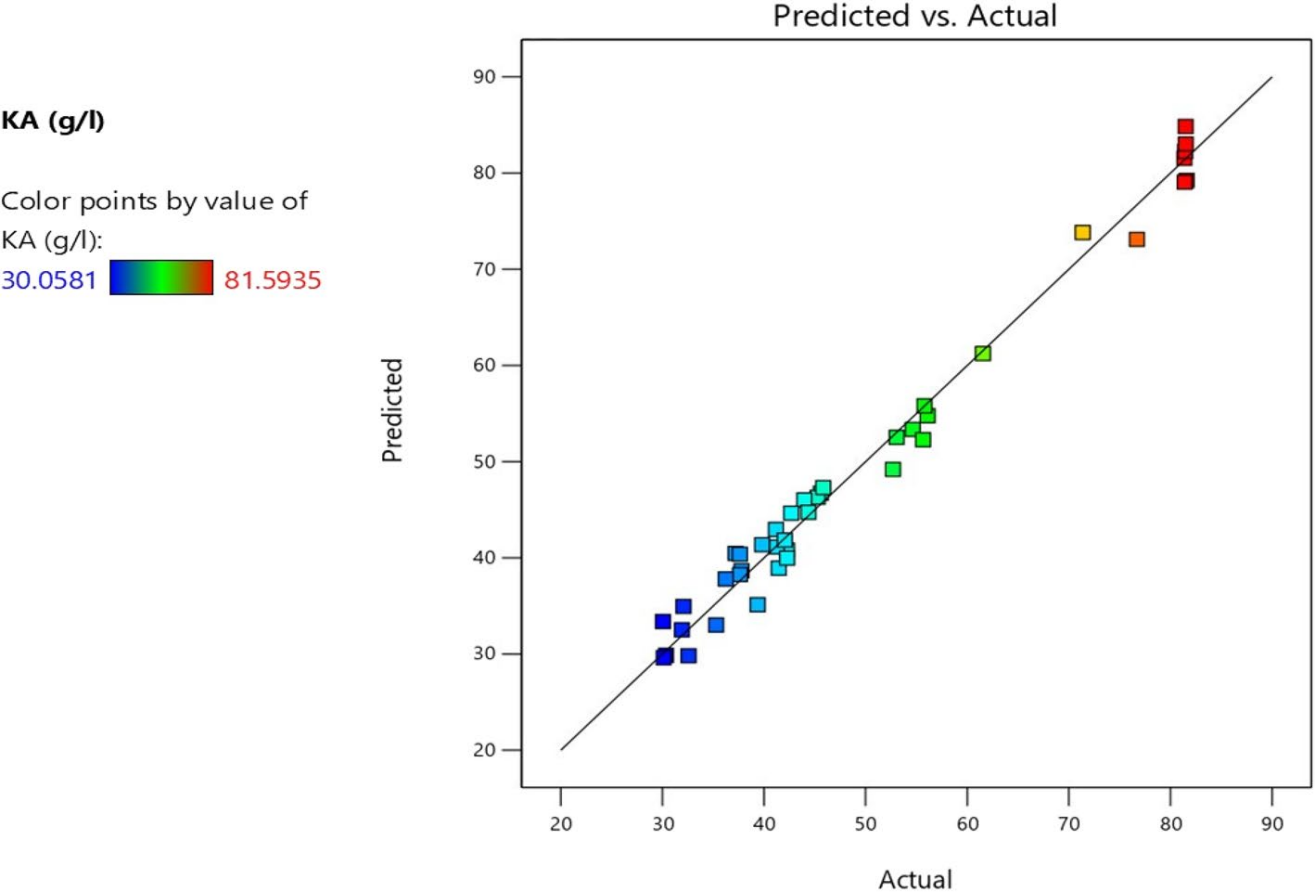
Actual (laboratory) and predicted (calculated) values of kojic acid production by *A. flavus* ASU45 (OL314748)

The maximum laboratory value of KA was 81.59 g/l; whereas the KA predicted value of 79.24 g/l was obtained in run number (24) using glucose 150, yeast extract 5, KH_2_PO_4_ 1, MgSO_4_.7H_2_O 2 g/l and pH 3 (Table S1). KA predicted values were found to be very near to the laboratory data, indicating the accuracy of the set model as cleared in Table S1 and Fig. 1. The statistical set model suitability, validity, significance, and accuracy were analyzed using one-way ANOVA as established in Table (S2). The ANOVA *F* and *P-* values of the KA set model was *F;* 63.32 and *P*; <0.0001 indicating the results significance at probability ≤0.05. Statistical coefficient (*R*^*2*^) was also assessed to clear the model goodness, accuracy, and fitting; *R*^*2*^ values of kojic acid production (g/l) was 98.45% and adjusted *R*^*2*^ value was 96.89% indicating that all set variations were illustrated significantly via the whole statistical model.

**Fig. 2:**
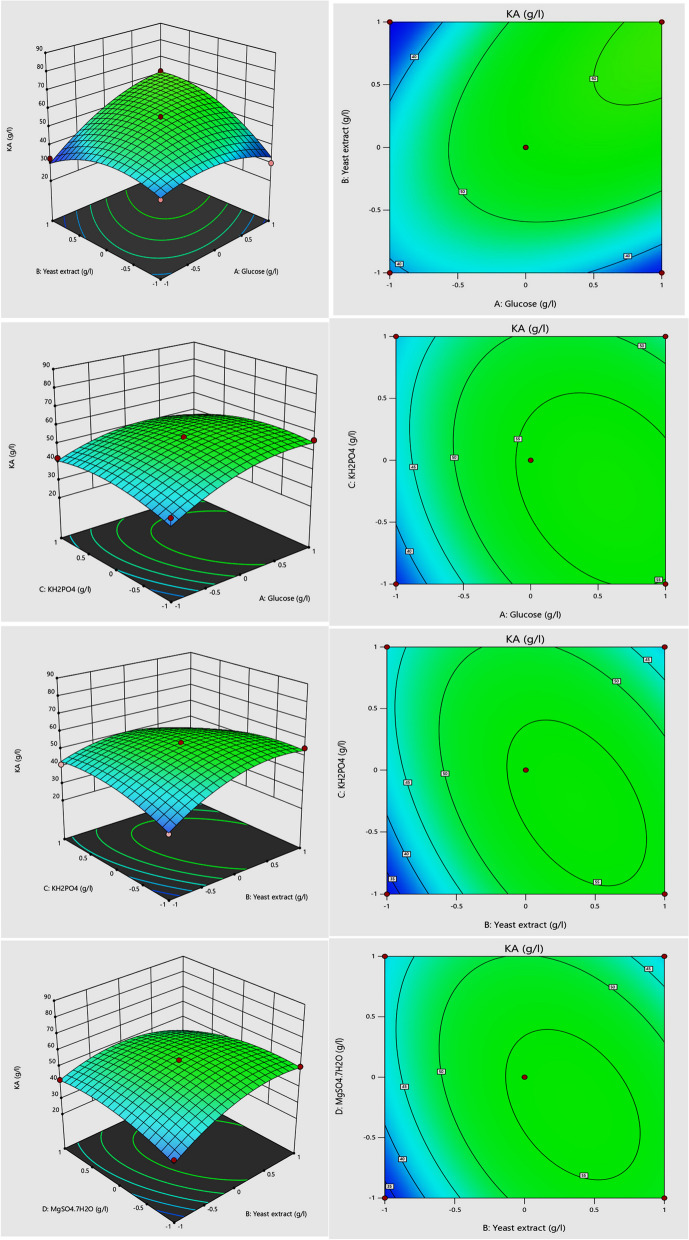
Box–Behnken statistical design 3D surface plots clearing the interactions between of AB (glucose * yeast extract), AC (glucose * KH_2_PO_4_), BC (yeast extract * KH_2_PO_4_), and BD (yeast extract * MgSO_4_.7H_2_O) on kojic acid (g/l) production by *A. flavus* ASU45 (OL314748)

**Fig. 3:**
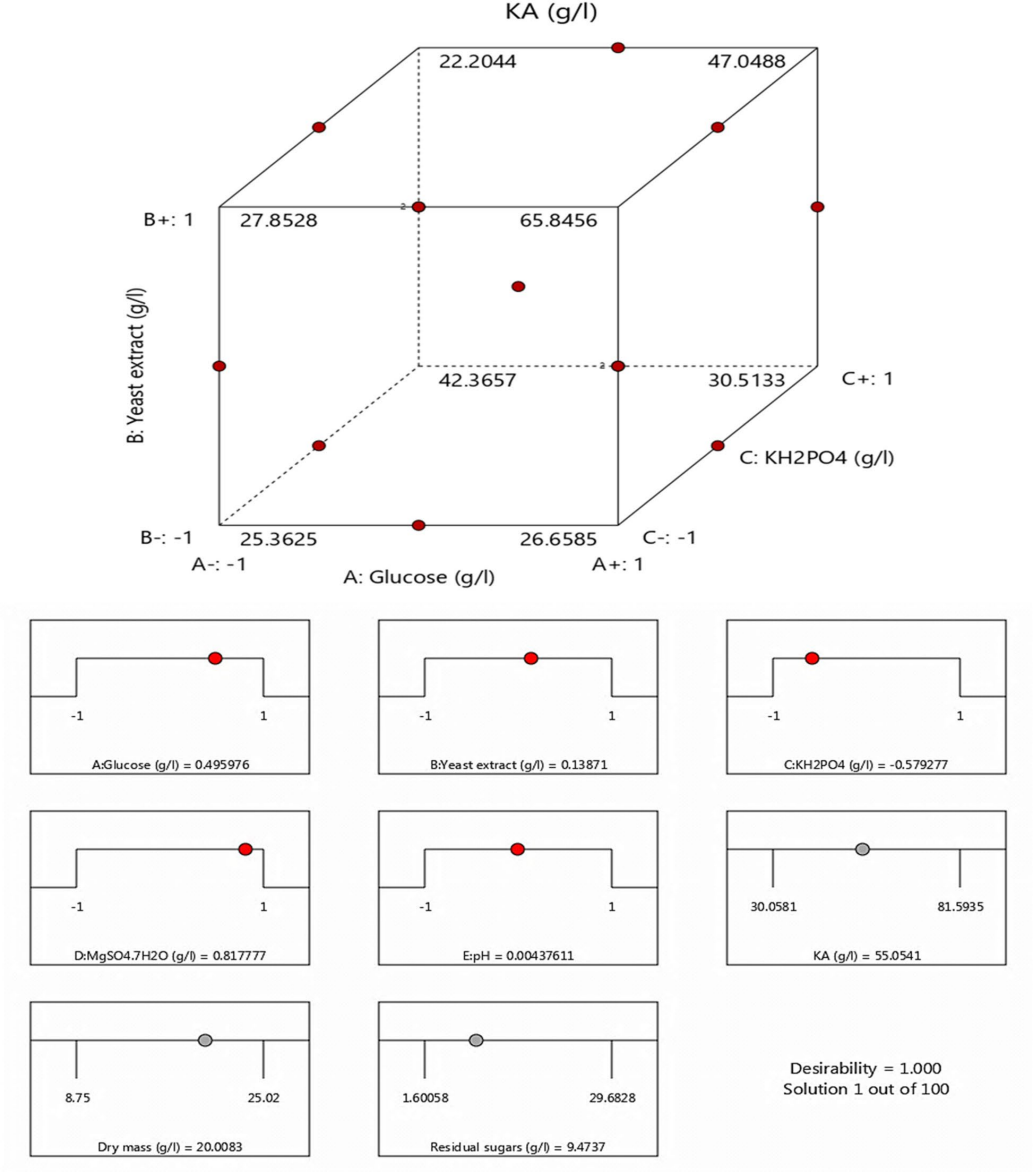
The statistical desirability ramp plot of kojic acid production by *A. flavus* ASU45 (OL314748)

Individual variables of glucose (A), yeast extract (B), and pH (E) have significant effects on kojic acid production, while KH_2_PO_4_(C), and MgSO_4_.7H_2_O (D) were non-significant. The interaction between different variables AB (glucose * yeast extract), AC (glucose * KH_2_PO_4_), BC (yeast extract * KH_2_PO_4_), and BD (yeast extract * MgSO_4_.7H_2_O) were significant for KA production. The response surface and the interaction plots were drawn in 3D visualization to clear the interaction between two factors in the constant of the other (Fig. 2). Derringer’s desirability function was used to calculate an optimal variable concentration for high KA production. The resulting optimum levels of glucose 150, g/l; yeast extract 5 g/l; KH_2_PO_4_ 1 g/l; MgSO_4_.7H_2_O 2 g/l, and pH 3, were found to be the best levels after using the function, giving desirability of 1.000 as cleared in Fig. 3.

### Characterization of kojic acid

**Fig. 4:**
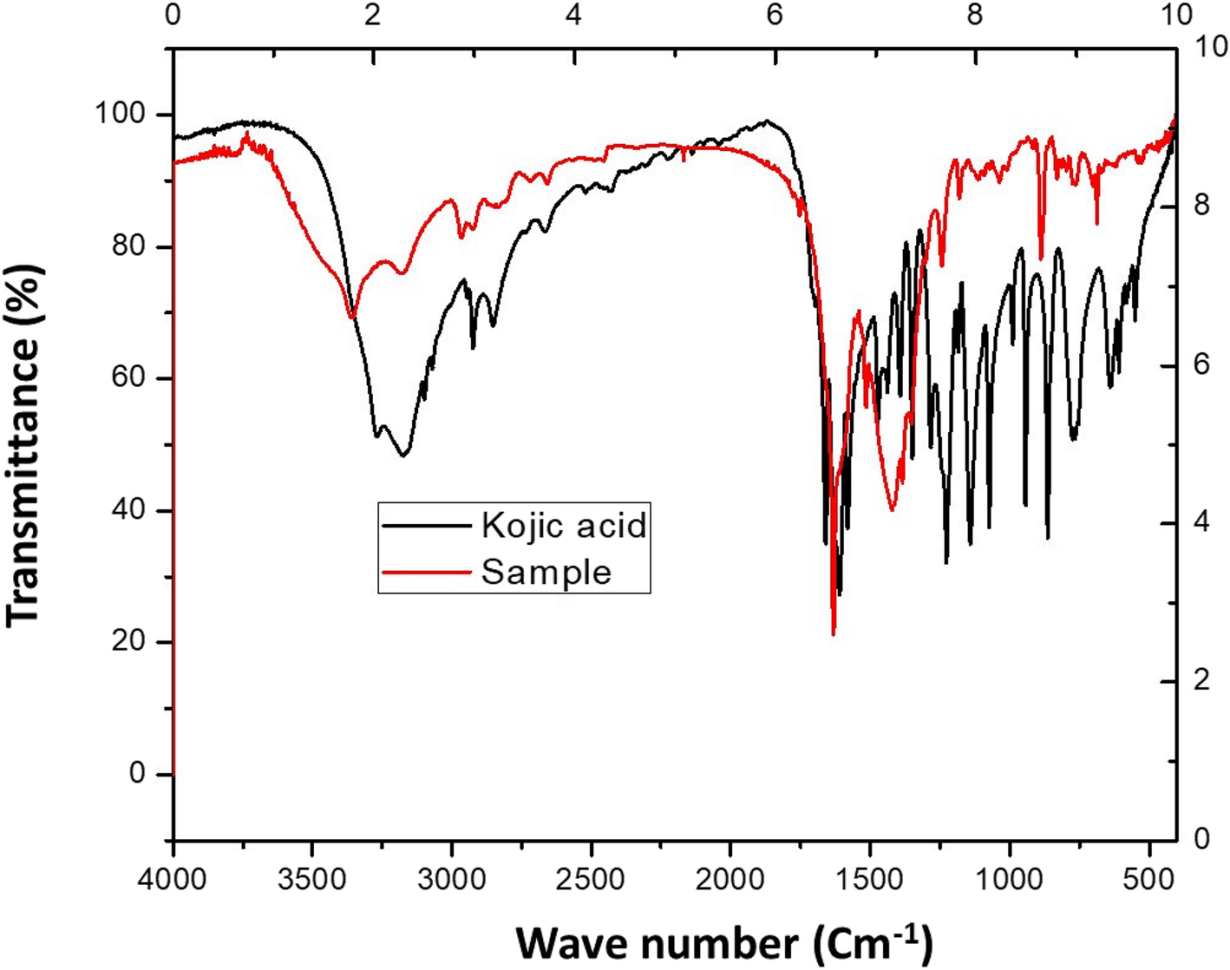
FTIR of standard and crystalized KA produced by *A. flavus* ASU45 (OL314748)

Kojic acid crystals were analyzed using FTIR, XRD, and a scanning electron microscope. FTIR spectrum of KA showed functional groups peak values similar to the standard KA sample. The functional group bands appear at 3176.55 cm-^1^ (for -OH), 2924.45, 2853.74 cm-^1^ (for aliphatic-CH), 1659.82 cm-^1^ (for cyclic -C=O), 1609.71 cm-^1^ (for C=C), 1471.64 cm-^1^ (for -CH_2_), 1073.56 cm-^1^ (for cyclic C-O-C), 943.07, 863.58, 764.99 cm-^1^ (for 1,4α-disubstituted ring). The standard KA has peaks at 3269.14, 3176.59 cm-^1^ (for -OH), 2924.40, 2852.55 cm-^1^ (for aliphatic-CH), 1660.55 cm-^1^ (for cyclic -C=O), 1610.02 cm-^1^ (for C=C), 1472.33 cm-^1^ (for -CH_2_), 1073.82 cm-^1^ (for cyclic C-O-C), 943.43, 863.54, 775.52 cm-^1^ (for 1, 4 α-disubstituted ring) as cleared in Fig. 4.

**Fig. 5:**
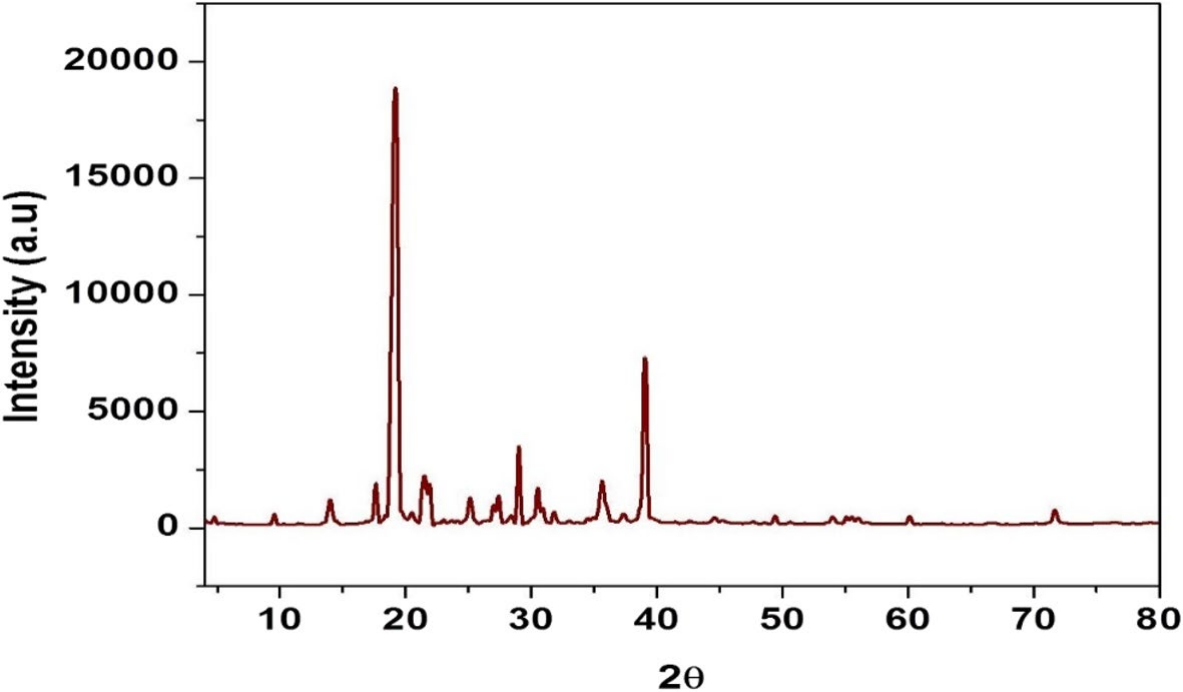
XRD of crystalized KA produced by *A. flavus* ASU45 (OL314748)

**Fig. 6:**
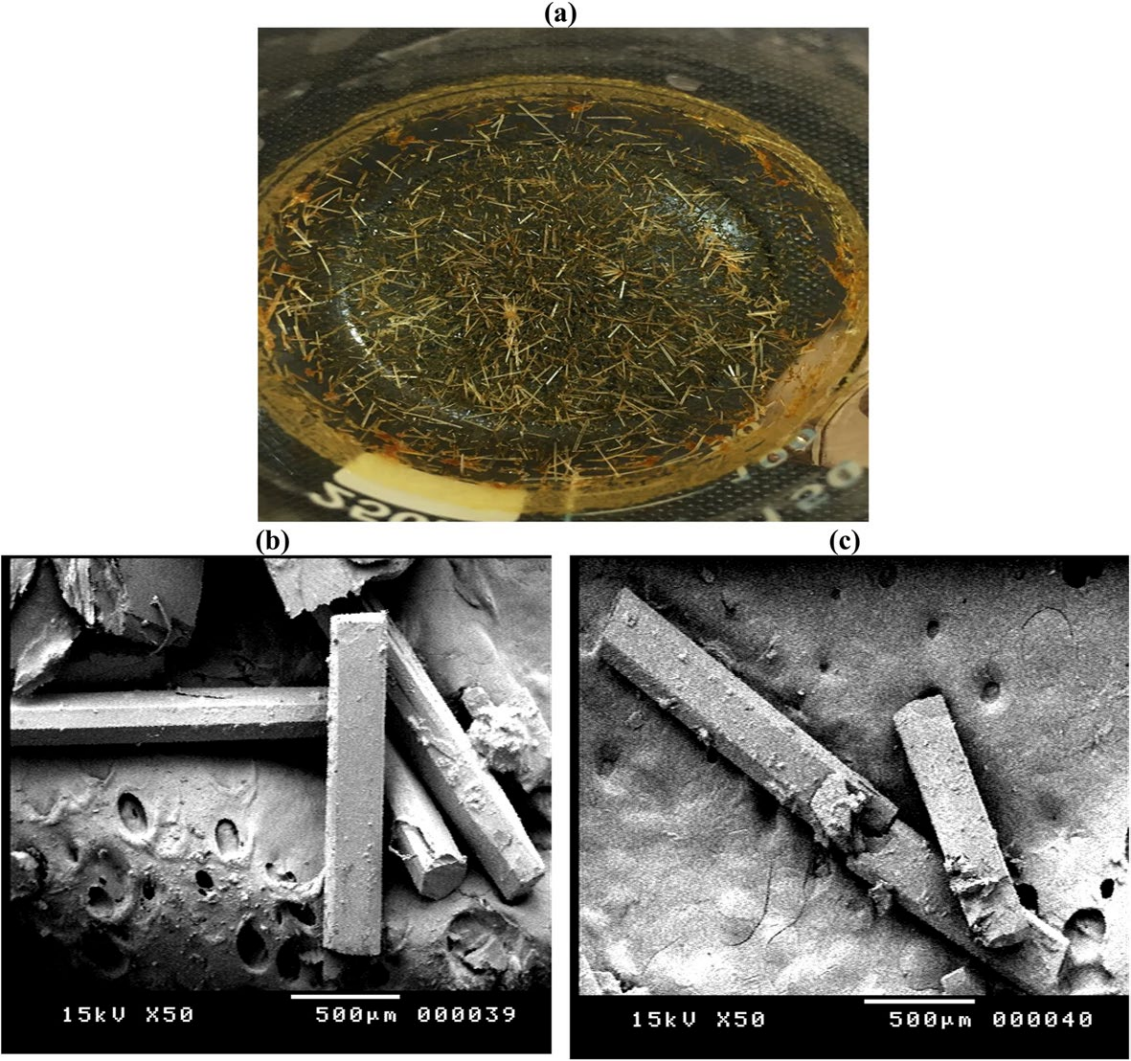
Crystalized KA produced by *A. flavus* ASU45 (OL314748) (**a**) under visible light, and (**b **& **c**) under scanning electron microscope

X-ray diffractogram of the extracted kojic acid is recorded and displayed in Fig. 5. This analysis depends on the elastic scattering of the X-rays from the analyzed structures, results reveal the presence of diffraction lines at 2θ = 17.6, 19.2, 21.6, 21.9, 29.0 and 39.1°, which match these lines of standard kojic acid identified card in JCPDS card No (00–007-0704) confirmed the presence of kojic acid. The crystal structure of KA was demonstrated using single crystal analysis in a previous study [48], with crystal size 0.1× 0.03× 0.02 (mm), with angles a/Å 3.76 (α/°), b/Å 18.34(β/°), 96.833(c/Å), and θ range 3.285–30.503. The crystalized structure of extracted kojic acid was cleared in Fig. 6 a-c. Kojic acid crystals appeared as yellow needles in visual light (Fig. 6a), using a scanning electron microscope KA crystal appeared as angular needles with 2000–2500 μm length and 250–300 μm width cleared in Figs. 6 b-c.

### Antibacterial activity of crystallized KA

The crystalized KA (cr. KA) was screened for their capabilities as antibacterial agents against six pathogenic bacteria *B. cereus* ASU300*, S. aureus* ASU301, *E. coli* ASU302*, K. pneumonia* ASU303*, S. marcescens* ASU304*,* and *S. plymuthica* ASU305 compared with standard KA (st. KA). It was clear that crystalized KA produced by *A. flavus* ASU45 (OL314748) was more effective than standard KA (chemically synthesized) against Gram +ve and -ve bacteria and its efficiency increased by increasing the concentration until 100 μg/ml, the most effective concentration (Table 1). *Bacillus cereus, K. pneumonia,* and *S. plymuthica* were the highest affected isolates at 100 μg/ml KA by 2.75 ± 0.05 (2.4 ± 0.1 st. KA), 2.85 ± 0.1 (2.55 ± 0.05 st. KA) and 2.85 ± 0.05 (2 ± 0.1 st. KA) inhibition zones cm ± SD, respectively. However, *E. coli*, *S. marcescens,* and *S. aureus* were inhibited by 2.35 ± 0.05 (2.15 ± 0.05 st. KA), 2.3 ± 0.1 (2.2 ± 0.1 st. KA) and 2.25 ± 0.05 (2.2 ± 0.0 st. KA) inhibition zones cm ± SD, respectively. For ethyl acetate (negative control), gave growth inhibition 1.4, 1.6, 1.5, 1.7, 1.5, 1.6 cm for *B. cereus* ASU300*, S. aureus* ASU301, *E. coli* ASU302*, K. pneumonia* ASU303*, S marcescens* ASU304*,* and *S. plymuthica* ASU305*.* Chloramphenicol was utilized as a positive control with 100 μg/ml concentration. Although chloramphenicol inhibited the bacterial growth, kojic acid gives higher antibacterial activities compared with chloramphenicol in 100 μg/ml concentration giving *B. cereus* ASU300 (1 ± 0.0 cm)*, S. aureus* ASU301 (1 ± 0.0 cm), *E. coli* ASU302 (1.4 ± 0.1 cm)*, K. pneumonia* ASU303 (1.1 ± 0.05)*, S. marcescens* ASU304 (1.8 ± 0.1 cm)*,* and *S. plymuthica* ASU305 (1.6 ± 0.05 cm).

**Table 1:** Antibacterial activities (inhibition zone (IZ), cm ± SD) of crystalized and standard kojic acid from *A. flavus* ASU45 (OL314748) against six human pathogenic bacteria

**KA μg/ml**	**KA-Standard**	**KA-Crystalized**	**KA-Standard**	**KA-Crystalized**	**KA-Standard**	KA-Crystalized
	***B. cereus*** **ASU300 (IZ, cm ± SD)**		***S. aureus*** **ASU301 (IZ, cm ± SD)**		***E. coli*** ASU302 (IZ, cm ± SD)	
100	2.4 ± 0.1	2.75 ± 0.05	2.2 ± 0.0	2.25 ± 0.05	2.15 ± 0.05	2.35 ± 0.05
75	2.2 ± 0.0	2.55 ± 0.05	2 ± 0.1	2 ± 0.0	2 ± 0.0	2.1 ± 0.1
50	2.05 ± 0.05	2.45 ± 0.05	1.9 ± 0.0	1.95 ± 0.05	1.85 ± 0.05	1.95 ± 0.05
25	1.8 ± 0.1	2.15 ± 0.15	1.75 ± 0.05	1.85 ± 0.05	1.7 ± 0.0	1.6 ± 0.0
**KA μg/ml**	***K. pneumonia*** **ASU303 (IZ, cm ± SD)**		***S. marcescens*** **ASU304 (IZ, cm ± SD)**		***S. plymuthica*** **ASU305 (IZ, cm ± SD)**	
100	2.55 ± 0.05	2.85 ± 0.1	2.2 ± 0.1	2.3 ± 0.1	2 ± 0.1	2.85 ± 0.05
75	2.35 ± 0.05	2.75 ± 0.05	2.1 ± 0.0	2.1 ± 0.0	1.85 ± 0.05	2.2 ± 0.1
50	2.3 ± 0.0	2.4 ± 0.1	1.95 ± 0.05	1.95 ± 0.05	1.7 ± 0.0	1.8 ± 0.0
25	2.15 ± 0.05	2.35 ± 0.05	1.7 ± 0.0	1.75 ± 0.05	1.55 ± 0.05	1.65 ± 0.05

*KA* kojic acid, *IZ* inhibition zone, *SD* standard deviation, *Bacillus cereus* ASU300, *Staphylococcus aureus* ASU301, *Escherichia coli* ASU302, *Klebsiella pneumonia* ASU303, *Serratia marcescens* ASU304, and *Serratia plymuthica* ASU305

### Anticancer activity of crystallized KA

#### Cell viability by MTT assay

**Fig. 7:**
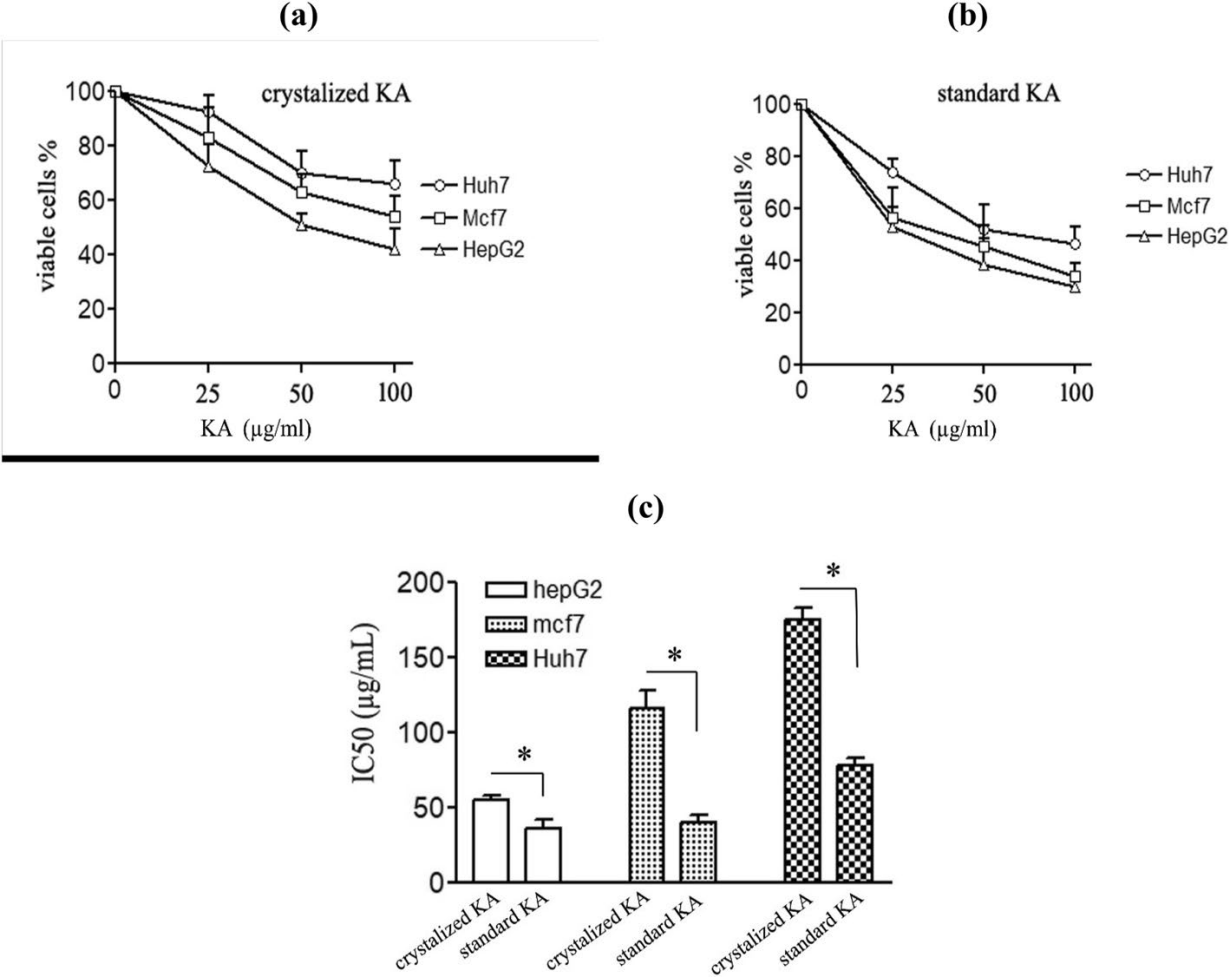
The effect of crystalized and standard Kojic acid on cell viability of different cell lines by MTT assay. Cells were treated with 0, 25, 50 and 100 μg/ml KA concentrations for 24 h and then cell viability was determined by MTT assay for both crystalized (**a**) and standard (**b**) kojic acid. IC_50_ of both forms of kojic acid was determined for 24 h (**c**). All data are means (*n* = 3) of independent experiments ± SD (* *p* < 0.05)

The toxicity of crystalized and standard KA toward different three cancer cell lines (HepG2, Mcf7, and Huh7) was examined after incubation for 24 h using 0, 25, 50, and 100 μg/ml KA concentrations. Cell viability was examined via MTT assays (Fig. 7a&b). The reduction of cell viability was dose-dependent. Among the examined cell lines, the most affected one was HepG2 followed by Mcf7 and Huh7. The IC_50_ of both crystalized and standard kojic acid was determined for 24 h on HepG2 cells (Fig. 7c), it was 55.1 ± 3.3 and 38.9 ± 6.2 μg/ml, respectively. These results are all in line with each other’s (Fig. 7a&b).

#### Acridine Orange/ethidium bromide staining assay

**Fig. 8:**
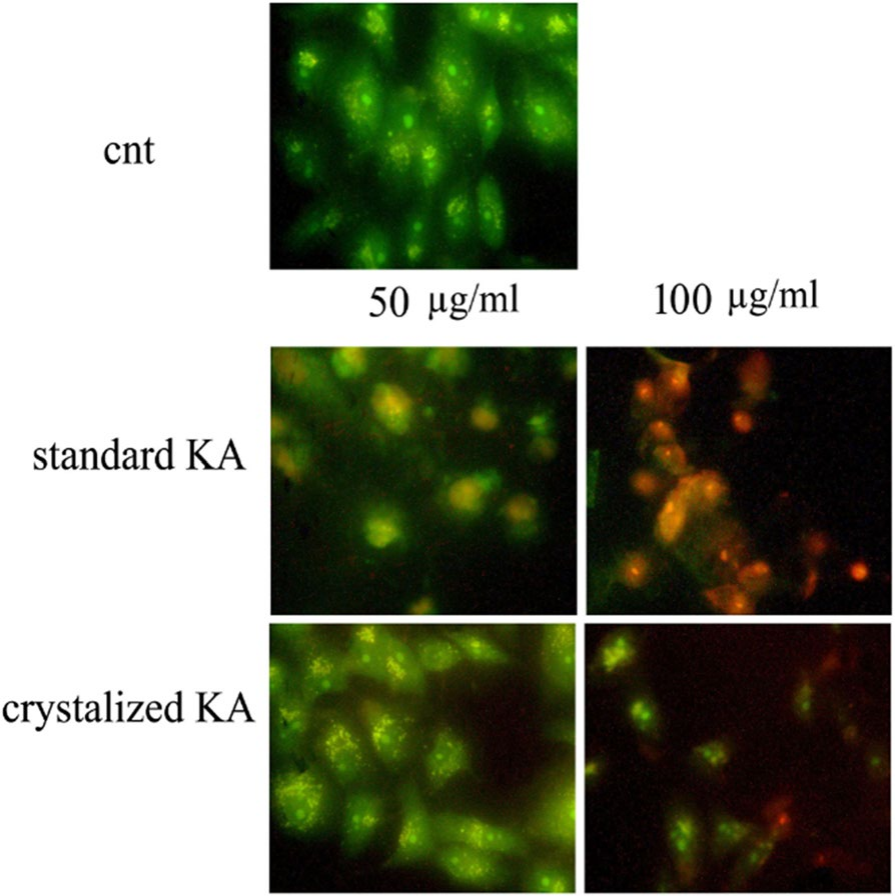
Examination of cancer cell death caused by kojic acid using AO/EB double staining HepG2 cell line transact with 0, 50, 100 μg/ml of crystalized and standard kojic acid for 24 h.Viable cells show bright green nuclei and intact structure; apoptotic cells clear orange nuclei and condensed chromatin; and the necrotic cells display orange nuclei and intact structure

The HepG2 cell death (the most affected cell line) was also examined morphologically by AO/EB double staining, to clear the percentage of apoptotic cells (pre- and late stages) with orange to red nuclei and with different degrees of chromatin damage increased in a dose-dependent manner for both crystalized and standard kojic acid (Fig. 8). The presence of necrotic cells was not evident across kojic acid concentrations. Clearly, effects of both crystalized and standard kojic acid on cell death were observed (Fig. 8).

#### Scratch assay

**Fig. 9:**
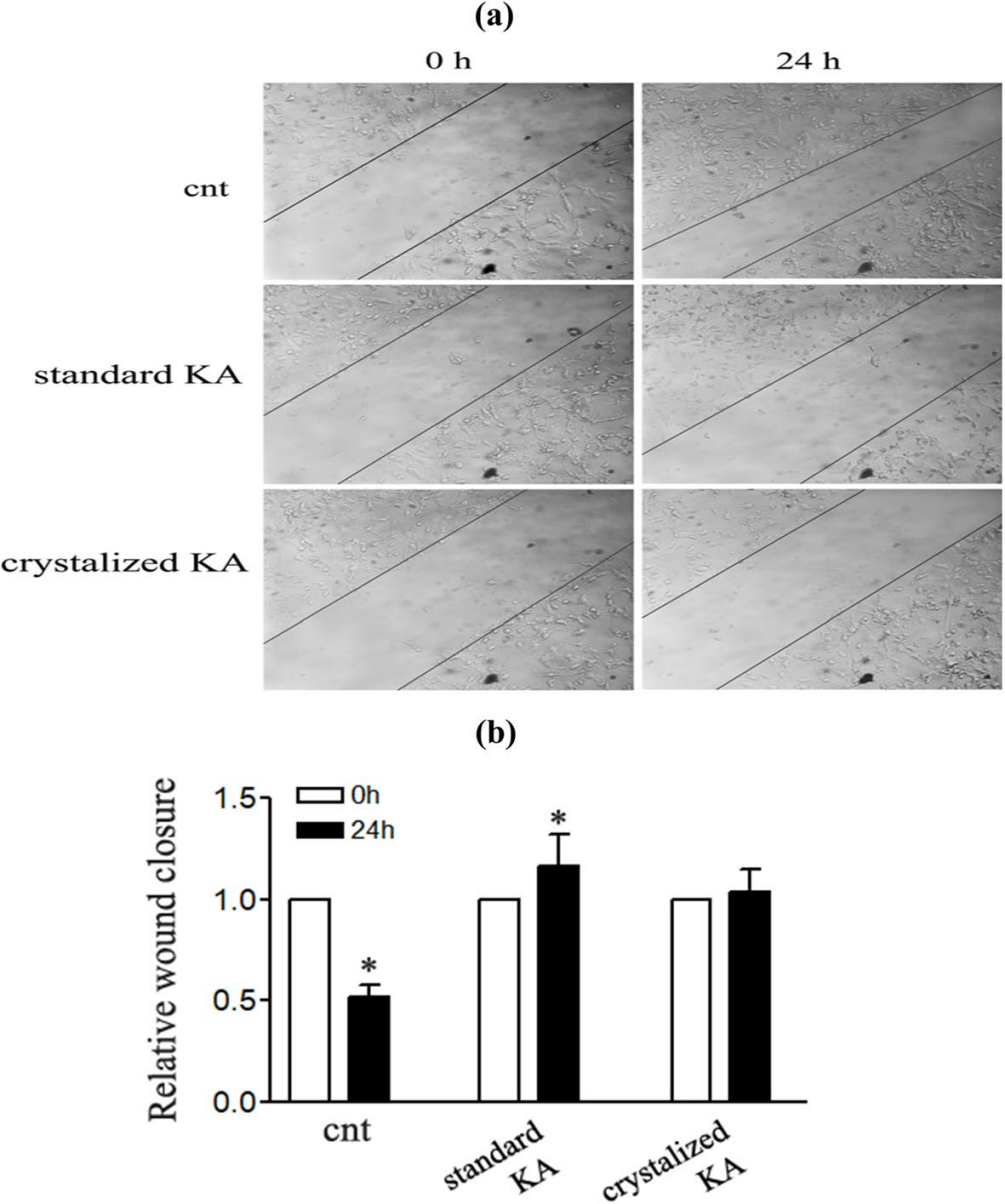
The ability of kojic acid treatment on cancer cell growth by scratch healing assay (HepG2) was plated for 24 h and then a scratch was carried out as described in methods section. Then cells treated with 0 and 50 μg/ml of crystalized and standard kojic acid for 24 h, the width of the scratch was determined at 0 and 24 h. of incubation. Representative images of wound closure of different treatments (**a**). Calculations of wound closure width after 0 and 24 h of different treatments (**b**). All data are means (*n* = 3) of independent experiments ± SD at *p* < 0.05

In another experiment, we used a scratch healing assay to test wound closure rate as an indication of cell growth. HepG2 cells were transacted or not with crystalized and standard kojic acid 50 μg/ml for 24 h, then the width of the scratch was determined at 0 and 24 h of incubation. The untreated cells have the smallest wound closure width (mm) compared to other treatments (Fig. 9a&b) which reflected the high inflammation rate of HepG2, while both crystalized and standard kojic acid had high wound closure width after 24 h incubation compared to the zero-hour incubation which clearing the anti-inflammation activities of the treatment. These results included that kojic acid is effective in cell growth amelioration.

### Molecular docking of KA as antibacterial and anticancer agent

#### As tyrosinase inhibitor

**Fig. 10:**
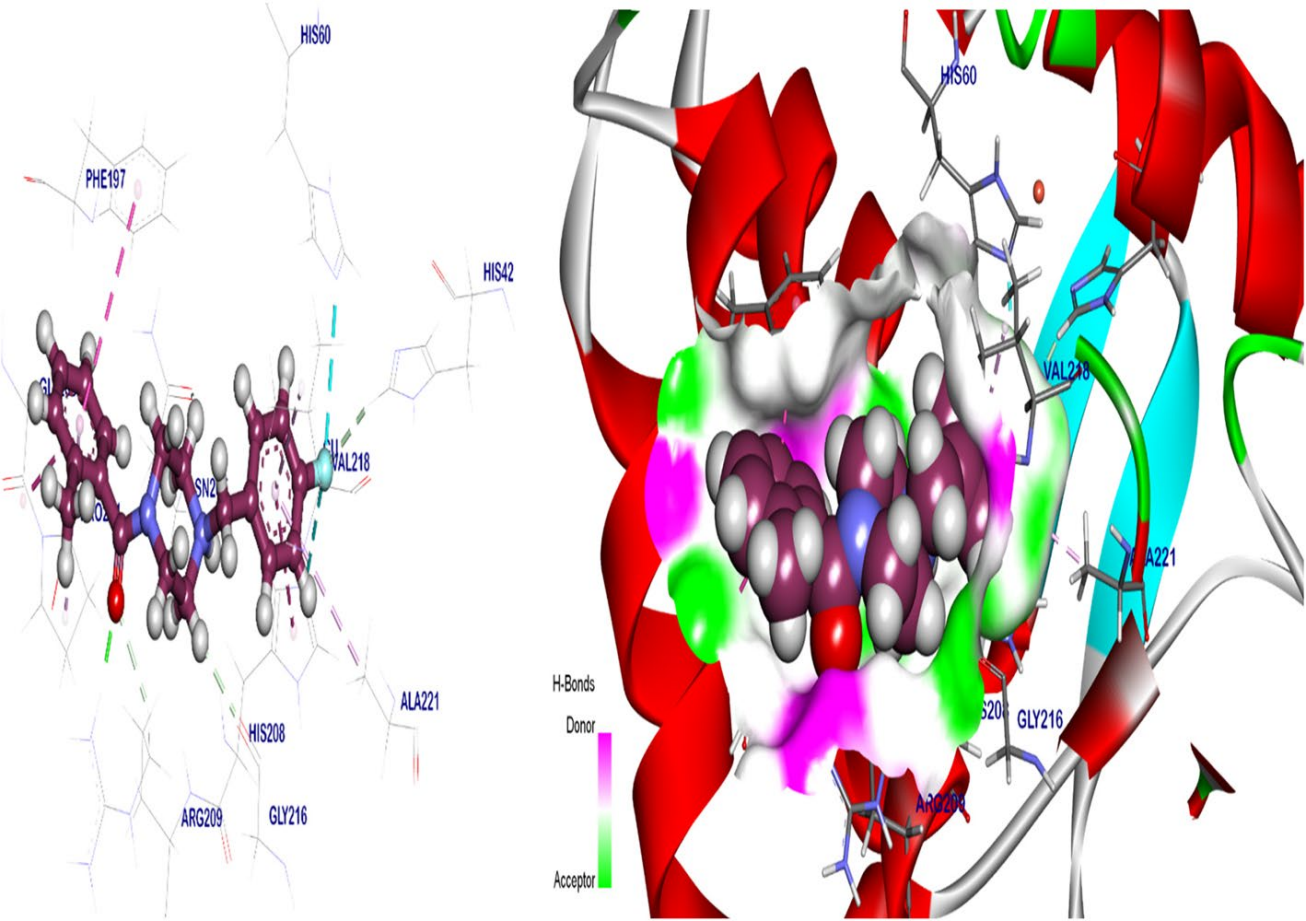
Co-crystalized ligand (B5N) docked in the Crystal Structure of tyrosinase*,* are in green color and the pi interactions are in purple lines with mapping surface clearing the co-crystalized ligand in the active pocket of tyrosinase crystal structure

**Fig. 11:**
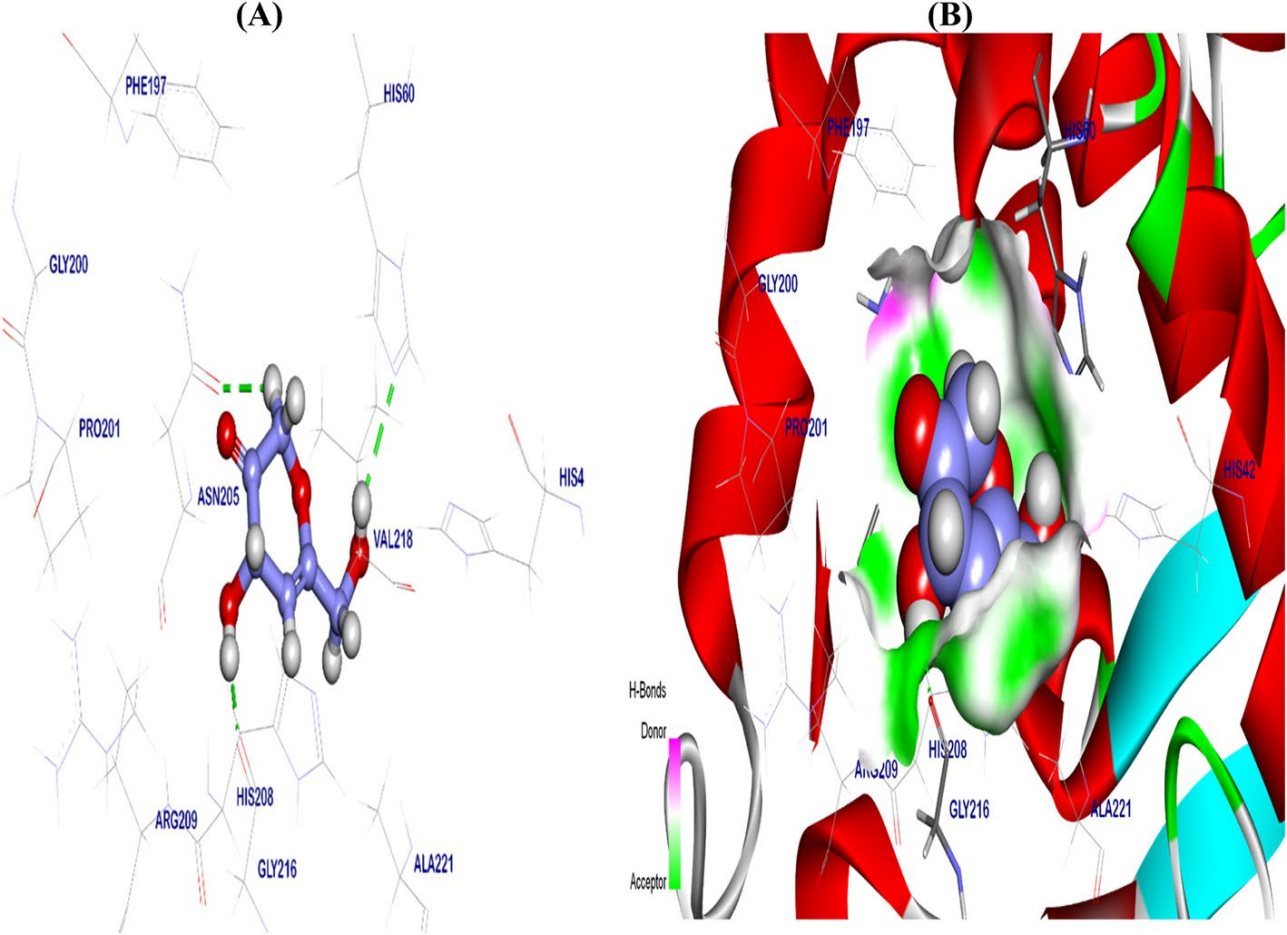
3D kojic acid docked in crystal structure of tyrosinase, hydrogen (H) bonds are in green color and the pi interactions are in purple lines (**A**), mapping surface clearing kojic acid occupying through the active pocket of tyrosinase crystal structure (**B**)

The co-crystalized ligand B5N binding mode exhibited − 6.56 kcal/ mol binding energy against the crystal structure of tyrosinase. Which binding with Arg209 by 1 H bond with 1.81 Å distance, moreover formed six Pi-amide, Pi-Alkyl, and Pi-Pi interactions with His208, Phe197, Gly200, Pro201, Val218, and Ala221. However, the Fluro group interacted with cupper 303 by metal interaction, His60, and His208 by halogen interaction (Fig. 10). The best pose of Kojic acid exhibited − 6.98 kcal/ mol binding energy against the crystal structure of tyrosinase. It’s binding with Gly216, His60 and Asn205 by 3 H bonds with 2.03, 2.56, and 2.51 Å distance, respectively, additionally interacted with cupper 303 by metal interaction (Fig. 11 & Table 2).

**Table 2:** Tested compounds DG (kcal/mol) against *the* tyrosinase crystal structure and NFK_b_ crystal structure via target sites PDB (ID: 6EI4) and (ID:5T8P), respectively

Ligand	RMSD value (Å)	Docking score (kcal/mol)	Interactions	
			**H.B**	Pi-interactions
**Tyrosinase crystal structure**				
**Crystal ligand (B5N)**	1.22	−6.56	1	6
**Kojic acid**	1.12	−6.98	3	0
**NFK**_**b**_ **crystal structure**				
**Crystal ligand (benzoxepine)**	0.66	−5.71	2	12
**Kojic acid**	0.89	−5.49	3	2

#### As nuclear factor kappa B (NFKb) inhibitor

**Fig. 12:**
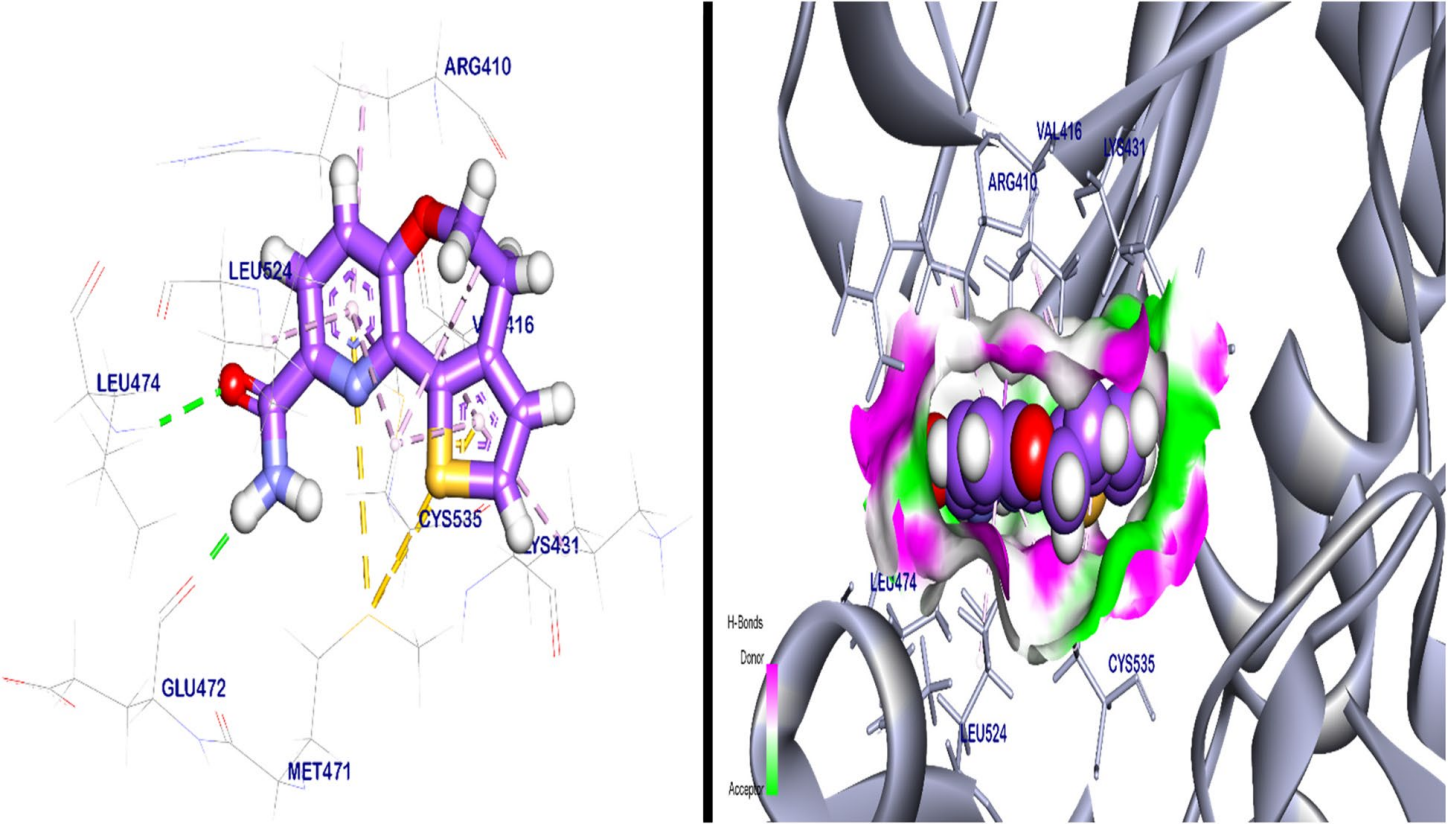
Co-crystalized ligand (benzoxepine) docked in the Crystal Structure of NFK_b_, hydrogen (H) bonds are in green color and the pi interactions are in purple lines with mapping surface clearing the co-crystalized ligand in the active pocket of NFK_b_ crystal structure

**Fig. 13:**
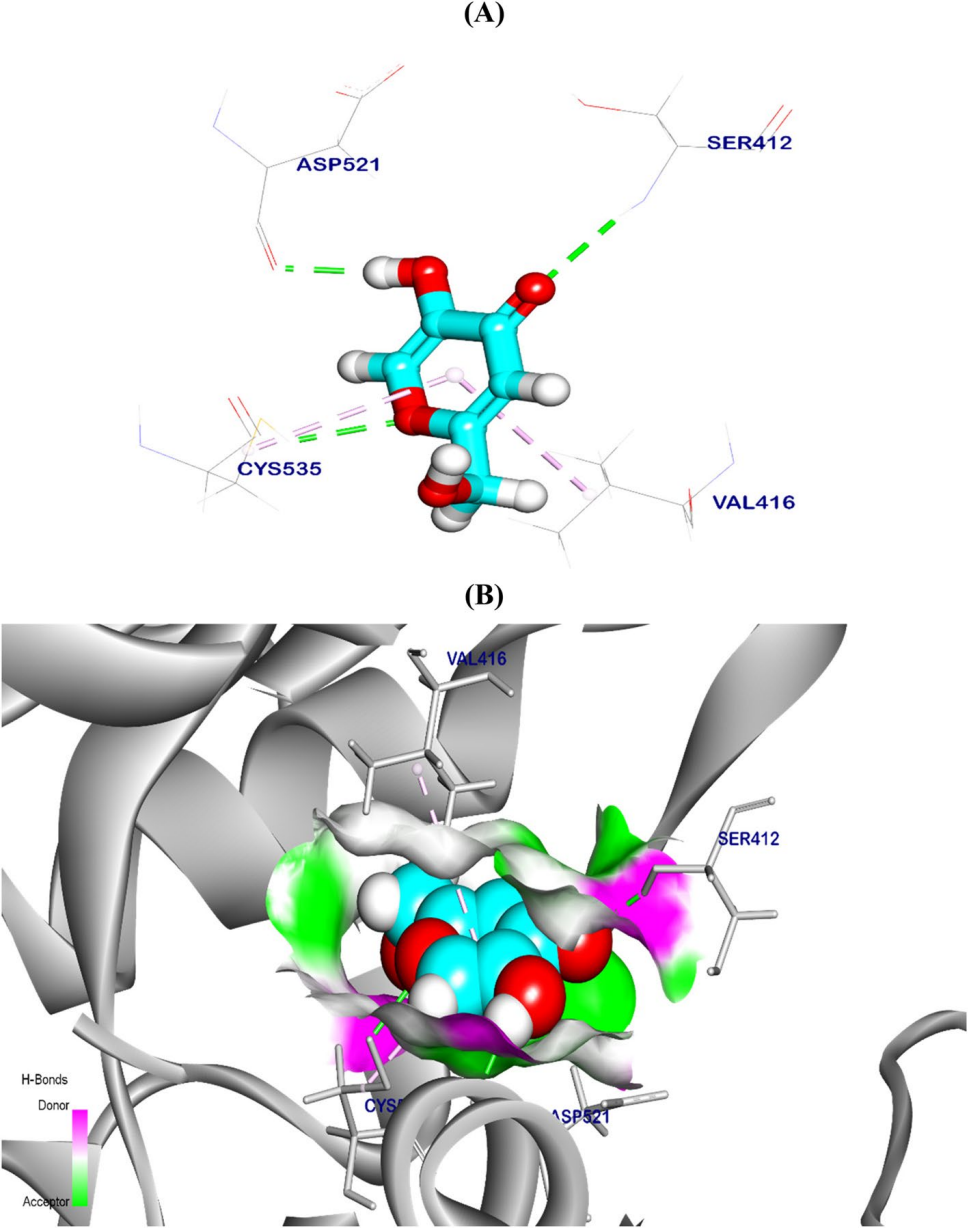
3D kojic acid docked in crystal structure of NFK_b_, hydrogen (H) bonds in green color and the pi interactions are in purple lines (**A**), mapping surface clearing kojic acid occupying through the active pocket of NFK_b_ crystal structure (**B**)

The binding mode of a co-crystalized ligand (benzoxepine) exhibited a binding energy of − 5.71 kcal/ mol against the Crystal Structure of NFK_b_. Which interacted with Leu474 and Glu472 by two hydrogen bonds with a distance of 2.50 and 1.87 Å, moreover, it’s formed 12 Pi-Alkyl, Pi-sigma, and Pi-sulfur interactions with Val416, Leu524, Arg410, Met471, Cys535, and Lys431 (Fig. 12). The best pose of KA exhibited a binding energy of − 5.49 kcal/ mol against the Crystal Structure of NFK_b_. It interacts with Asp521, Cys535, and Ser412 by three hydrogen bonds with a distance of 2.13, 2.51 and 2.71 Å respectively, and additionally interacted with Val416 and Cys535 by Pi-Alkyl interaction (Fig. 13).

## Discussion

Kojic acid represents a secondary biological metabolite produced mostly by *Aspergillus* spp. In the first part of the study, we enhanced KA production by *A. flavus* ASU45 (non-aflatoxin producer) from 39.96 to 81.59 g/l KA using Box-Behnken statistical design in modified Czapek’s glucose broth medium. Several researchers demonstrated the ability of fungi for KA production; Machida et al. [58] revealed that *Aspergillus oryzae* and *A. flavus* were the main producers of kojic acid. Mahmoud and Zohri [17] reported that KA produced by different isolates of *A. flavus* and *A. oryzae* from Egyptian sources were between 0.091 and 66.18 g/l on a glucose medium. It was found that *A. oryzae* could produce 24 to 41 g/l KA in a glucose medium [15, 59], however, Yan et al. [53] obtained 33.1 g/l KA from *A. oryzae* M866 in a corn stalk medium. Also, it was found that *A. flavus* could produce 24 to 47 g/l KA using glucose as a carbon source [12, 13, 52, 60–64].

Statistical experimental designs were recently preferred in the optimization process over the traditional way (one factor at a time) for saving time, costs, minimizing the experimental errors, and showing the interaction between the tested parameters [17]. After optimizing KA production by *A. flavus* ASU45 (OL314748) using Box-Behnken statistical design, kojic acid production values increased to 81.59 g/l (with predicted value 79.24 g/l) using glucose 150, yeast extract 5, KH_2_PO_4_ 1, MgSO_4_.7H_2_O 2 g/l and pH 3. In agreement with our findings; after the optimization process of KA from *A. flavus* the researcher reached varied quantities; Zohri et al. [12] obtained 24.65 g/l KA, Sanjotha et al., [13] obtained 31 g/l KA, Rosfarizan and Ariff [64] obtained 39.9 g/l KA, Devi et al. [63] obtained 47 g/l KA, El-Kady et al. [11] obtained 53.5 g/l KA, and Devi et al. [65] obtained 82.6 g/l of kojic acid. In our study, the kojic acid was extracted and crystalized using ethyl acetate (1:1, filtrate: solvent). Previous researchers reported ethyl acetate is the best extraction solvent for kojic acid and the equal ratio is the effective extraction solution [62, 66]. Crystallized KA was characterized using FTIR and XRD showing high agreement with previously reported results by Devi et al. [67].

In the second part, we compare the antibacterial activities of crystalized (from *Aspergillus flavus* ASU45) and standard (chemically synthesized) in the presence of chloramphenicol as positive control. Crystalized KA was an effective antibacterial agent against all the six pathogenic bacteria; *B. cereus* ASU300*, S. aureus* ASU301, *E. coli* ASU302*, K. pneumonia* ASU303*, S. marcescens* ASU304*,* and *S. plymuthica* ASU305. However, *B. cereus, K. pneumonia,* and *S. plymuthica* were the highest inhibited isolates. In agreement with our finding; KA was recorded as an antimicrobial agent to both Gram-ve and + ve bacteria [68] with dilutions from 1:1000 to 1:2000 [38]. *Bacillus* sp., *E. coli* and *S. aureus* were controlled effectively by KA [3, 41], and the concentrations about or higher than 100 ppm were the effective ones [69, 70]. It was demonstrated that 100 μg/ml KA was an effective antibacterial concentration against *Escherichia coli, Klebsiella pneumoniae, Staphylococcus aureus, Pseudomonas oleovorans* and *Staphylococcus epidermidis* which was in agreement with our findings [71–73]. Kojic acid also demonstrated efficient antibacterial activity to *Acinetobacter baumannii* with MIC 128 μg/ml [74], *Micrococcus luteus* with 0.125 mg/ml [75], *Bacillus sphericus* with 200 μg/ml [71], *Bacillus subtilis* with 131.12 μM [72], 200 μg/ml [71], and *Streptococcus pyogenes* with 64 μg/ml [76]. Compared with common synthetic antibacterial agents kojic acid reveals more efficiency than chloramphenicol, it was demonstrated that chloramphenicol MIC > 256 μg/mL for *Escherichia coli, Providencia stuartii, Enterobacter cloacae,* and *Pseudomonas aeruginosa* [77] 128 μg/mL for *Enterobacter aerogenes*, and *Klebsiella pneumoniae* [78].

Kojic acid was found to exert its antibacterial activity near the cell surface; however, it was believed that the antibacterial action of kojic acid was related to metal ions chelation [79]. According to Wu [80] kojic acid -CH2OH group at position 2 is utilized as a binding site for attaching to bacterial surface then KA induces membrane perforation in bacteria leading to cytoplasmatic leakage, functions and bacterial cell death. Tyrosinase inhibitors like KA could be promising antimicrobials for enhancing the activity of the incumbent drugs. Recently, tyrosinase inhibition involved as an antibacterial mechanism depending on the cell membrane disruption [81]. Scientists observed strong antibacterial activity with strong tyrosinase inhibition, the antibacterial mechanism of tyrosinase inhibitors generated from the reduction of cellular membranes fluidity, which interferes with the bacterial function [82–84]. In our research, we illustrated the antibacterial mechanism of KA using the molecular docking technique as a tyrosinase inhibitor. The molecular docking process illustrates the molecule’s behavior through the binding sites of the target proteins via simulating the interaction process between the molecule and the protein through atomic levels [85]. Prediction of the ligand, location, orientation via these sites (referred as pose) and the evaluation of their binding affinity represents two fundamental processes through the docking process [86]. Essentially, the purpose of molecular docking is to use computational techniques to anticipate the structure of the ligand-receptor complex [87].

Both forms of kojic acid (standard and crystalized KA) were tested for the anticancer activities against three types of cancer cell lines; mammary carcinoma (Mcf-7), and hepatocellular carcinoma (HepG2, and Huh7). Both forms of kojic acid demonstrated anti-proliferative effect on the studied cell lines as tested by MTT assay. More detailed studies were done on HepG2 cell line, as acridine Orange/ ethidium bromide double staining for detection of cell death type and wound healing assay for investigation of cell growth. Reactive Oxygen Species (ROS) represent the primary source of diseases as cancer, Parkinson’s, Alzheimer’s, and many others [88]. Kojic acid has anti-oxidative abilities that neutralize ROS and accordingly protect against the development of such diseases [89]. It has been shown that KA has a potential antioxidant activity with a very close IC_50_ of ascorbic acid [42]. Accordingly, KA represents an effective non-toxic biological metabolite, that could neutralize the effects of ROS are produced through the chemotherapy strategies of neoplastic diseases [90]. In our study, kojic acid showed cytotoxic effects on the HepG-2 cells which suggest their strong antitumor potentiality against hepatocellular carcinoma. These results were in agreement with a previous study on chemically synthesized KA, which documented the combination therapy of Mannich base containing ciprofloxacin and Kojic acid in treating HepG-2 [43]. In our research, we illustrated the anticancer mechanism of KA using the molecular docking technique as a nuclear factor kappa B (NFK_b_) inhibitor. NFK_b_ represents the main transcription factors regulating the genes responsible for the inflammatory responses stimulating the immunoglobulin κ light chain expression in B cells [91]. Kojic acid was reported as a potential inhibitor of cellular NF- kappa B, HaCaT cells, SCC-13, and human keratinocytes [92].

kojic acid was found not toxic in reproductive, chronic, genotoxicity, and acute studies due to its slowly releasing properties throughout the human skin [93, 94]. The toxicity of kojic acid is connected basically with the concentration [93]. The concentrations utilized during the antibacterial and anticancer treatments didn’t exceed 100 μg/ml which was important to select the convenient concentrations for future applications. Although the effective biological properties of KA, its concentrations in human treatments were recommended to not exceed 1% for safety use [95], not exceed 4% for skin care and cosmetics [2], and not exceed 2% for face and neck (leave-on products) [96] which is much higher than the concentrations we used (the highest concentration was 0.01%).

### Future prospective

Kojic acid has various applications in several fields as antimicrobial, anticancer, tyrosinase inhibitor, insecticide and pesticide organic compounds. Applications of kojic acid in several fields as well as its market value are increasing frequently. Researchers and producers of kojic acid hope to search for new KA-producing microorganisms that have the natural adaptability on the way to industrial process conditions and to consume low-cost substrates for their growth that are positioned within technological advances. The preparation of natural kojic acid derivatives are promising and advantageous to apply in human or veterinary medicines and needs to include with preferable properties during future studies as a natural solution to drug resistance pathogens and even in cancer treatments.

## Conclusions

It concluded that statistical designs are effective and time-saving tools to optimize the production process and clear the interaction manner between tested parameters. Kojic acid was increased with double production using Box-Behnken statistical design with 41 runs and 98.45% efficiency. Fourier-transform infrared spectroscopy, X-ray diffraction, and scanning electron microscope are used for crystalized kojic acid analysis. Crystalized kojic acid has effective antibacterial activities against six human pathogenic bacteria *B. cereus* ASU 300, *S. aureus* ASU 301, *E. coli* ASU302, *K. pneumonia* ASU 303, *S. marcescens* ASU 304, and *S. plymuthica* ASU 305. Also, it has anticancer activities against Mcf-7, HepG2 and Huh7 cancer cell lines and showed high cytotoxic effects on HepG-2 cells which suggests its strong antitumor against hepatocellular carcinoma. The biological mechanisms of kojic acid as an antibacterial and anticancer agent were illustrated using the molecular docking technique.

### Supplementary Information

**Additional file 1: Table S1.** Box-Behnken design with five variables; glucose (A), yeast extract (B), KH2PO4 (C), MgSO4·7H2O (D) and pH (E) with actual and/or predicted responses of kojic acid (g/l) (KA) by Aspergillus flavus ASU45 (Accession no. OL314748); **Table S2.** ANOVA results for Box-Behnken quadratic model of kojic acid (g/l) by Aspergillus flavus ASU45 (Accession no. OL314748).

## Data Availability

The authors confirm that the data supporting the findings of this study are available within the article and indicated supplementary materials. Sequence data that support the findings of this study have been deposited in the NCBI with the accession code *Aspergillus flavus* ASU45 (Accession no. OL314748) (https://www.ncbi.nlm.nih.gov/nuccore/OL314732).
