# Supplementary material for: Production of kojic acid by Aspergillus flavus OL314748 using box-Behnken statistical design and its antibacterial and anticancer applications using molecular docking technique
Source: BMC Microbiol. 2024 Apr 25;24:140. doi: 10.1186/s12866-024-03289-2 (PMC11044385; doi:10.1186/s12866-024-03289-2)
Supplement: Supplementary file 1 — Additional file 1: Table S1. Box-Behnken design with five variables; glucose (A), yeast extract (B), KH2PO4 (C), MgSO4·7H2O (D) and pH (E) with actual and/or predicted responses of kojic acid (g/l) (KA) by Aspergillus flavus ASU45 (Accession no. OL314748); Table S2. ANOVA results for Box-Behnken quadratic model of kojic acid (g/l) by Aspergillus flavus ASU45 (Accession no. OL314748). [file 12866_2024_3289_MOESM1_ESM.docx]

| **Table S1: Box-Behnken design with five variables; Glucose (g/l) (A), yeast extract (g/l) (B), KH_2_PO_4_ (g/l) (C), MgSO_4_·7H_2_O (g/l) (D) and pH (E) with actual and/or predicted responses of kojic acid (g/l) (KA) by *Aspergillus flavus* ASU45 (Accession no.** [**OL314748**](https://www.ncbi.nlm.nih.gov/nuccore/OL314732)**).** | | | | | | | | |
| --- | --- | --- | --- | --- | --- | --- | --- | --- |
| **Trials** | **Glucose (g/l)** | **Yeast extract (g/l)** | **KH_2_PO_4_ (g/l)** | **MgSO_4_·7H_2_O (g/l)** | | **pH (E)** | **Actual values of KA (g/l)** | **Predicted values of KA (g/l)** |
| **1** | **1** | **-1** | **0** | **0** | | **0** | **30.06** | **33.38** |
| **2** | **0** | **1** | **0** | **0** | | **1** | **37.19** | **40.46** |
| **3** | **1** | **0** | **0** | **0** | | **1** | **44.37** | **44.74** |
| **4** | **0** | **1** | **1** | **0** | | **0** | **41.25** | **41.13** |
| **5** | **0** | **-1** | **0** | **1** | | **0** | **42.05** | **41.85** |
| **6** | **0** | **0** | **-1** | **1** | | **0** | **43.96** | **46.02** |
| **7** | **0** | **-1** | **0** | **0** | | **1** | **30.36** | **29.87** |
| **8** | **0** | **-1** | **1** | **0** | | **0** | **41.18** | **42.95** |
| **9** | **1** | **1** | **0** | **0** | | **0** | **61.54** | **61.24** |
| **10** | **-1** | **0** | **0** | **0** | | **-1** | **71.36** | **73.82** |
| **11** | **0** | **0** | **-1** | **-1** | | **0** | **45.59** | **46.71** |
| **12** | **0** | **1** | **0** | **-1** | | **0** | **53.05** | **52.53** |
| **13** | **0** | **0** | **1** | **0** | | **1** | **42.27** | **39.98** |
| **14** | **0** | **0** | **-1** | **0** | | **-1** | **81.50** | **83.03** |
| **15** | **-1** | **1** | **0** | **0** | | **0** | **32.57** | **29.82** |
| **16** | **0** | **-1** | **0** | **0** | | **-1** | **76.70** | **73.10** |
| **17** | **-1** | **0** | **0** | **-1** | | **0** | **37.63** | **40.38** |
| **18** | **1** | **0** | **0** | **-1** | | **0** | **52.70** | **49.19** |
| **19** | **0** | **1** | **0** | **1** | | **0** | **39.81** | **41.36** |
| **20** | **1** | **0** | **1** | **0** | | **0** | **45.83** | **47.30** |
| **21** | **0** | **0** | **0** | **-1** | | **-1** | **81.45** | **82.25** |
| **22** | **1** | **0** | **-1** | | **0** | **0** | **56.10** | **54.77** |
| **23** | **0** | **0** | **1** | | **0** | **-1** | **81.40** | **79.06** |
| **24** | **0** | **0** | **0** | | **1** | **-1** | **81.59** | **79.24** |
| **25** | **-1** | **0** | **-1** | | **0** | **0** | **39.37** | **35.13** |
| **26** | **0** | **1** | **-1** | | **0** | **0** | **54.64** | **53.36** |
| **27** | **0** | **0** | **0** | | **-1** | **1** | **37.64** | **38.26** |
| **28** | **-1** | **0** | **0** | | **1** | **0** | **32.07** | **34.95** |
| **29** | **0** | **-1** | **0** | | **-1** | **0** | **35.29** | **33.02** |
| **30** | **0** | **0** | **0** | | **1** | **1** | **41.45** | **38.92** |
| **31** | **0** | **0** | **1** | | **1** | **0** | **42.68** | **44.64** |
| **32** | **-1** | **0** | **0** | | **0** | **1** | **30.13** | **29.62** |
| **33** | **0** | **0** | **1** | | **-1** | **0** | **45.28** | **46.29** |
| **34** | **-1** | **-1** | **0** | | **0** | **0** | **37.79** | **38.66** |
| **35** | **1** | **0** | **0** | | **0** | **-1** | **81.50** | **84.84** |
| **36** | **-1** | **0** | **1** | | **0** | **0** | **42.26** | **40.80** |
| **37** | **0** | **-1** | **-1** | | **0** | **0** | **31.92** | **32.52** |
| **38** | **0** | **0** | **0** | | **0** | **0** | **55.80** | **55.80** |
| **39** | **0** | **0** | **-1** | | **0** | **1** | **36.24** | **37.80** |
| **40** | **1** | **0** | **0** | | **1** | **0** | **55.65** | **52.28** |
| **41** | **0** | **1** | **0** | | **0** | **-1** | **81.37** | **81.54** |

| **Table S2: ANOVA results for Box-Behnken quadratic model of kojic acid (g/l) by *Aspergillus flavus* ASU45 (Accession no.** [**OL314748**](https://www.ncbi.nlm.nih.gov/nuccore/OL314732)**).** | | | | |
| --- | --- | --- | --- | --- |
| **Source** | **Sum of Squares** | **Mean Square** | ***F*-value** | ***P-value*** |
| **Model** | **11164.74** | **558.24** | **63.32** | **< 0.0001** |
| **A-Glucose (g/l)** | **683.32** | **683.32** | **77.50** | **< 0.0001** |
| **B-Yeast extract (g/l)** | **361.98** | **361.98** | **41.06** | **< 0.0001** |
| **C-KH_2_PO_4_ (g/l)** | **3.22** | **3.22** | **0.3649** | **0.5526** |
| **D-MgSO_4_.7H_2_O (g/l)** | **5.48** | **5.48** | **0.6221** | **0.4395** |
| **E-pH** | **7107.85** | **7107.85** | **806.20** | **< 0.0001** |
| **AB** | **336.66** | **336.66** | **38.19** | **< 0.0001** |
| **AC** | **43.22** | **43.22** | **4.90** | **0.0386** |
| **AD** | **18.10** | **18.10** | **2.05** | **0.1673** |
| **AE** | **4.21** | **4.21** | **0.4774** | **0.4975** |
| **BC** | **128.27** | **128.27** | **14.55** | **0.0011** |
| **BD** | **99.94** | **99.94** | **11.34** | **0.0031** |
| **BE** | **1.16** | **1.16** | **0.1317** | **0.7205** |
| **CD** | **0.2341** | **0.2341** | **0.0266** | **0.8722** |
| **CE** | **9.41** | **9.41** | **1.07** | **0.3139** |
| **DE** | **3.36** | **3.36** | **0.3808** | **0.5441** |
| **A²** | **131.09** | **131.09** | **14.87** | **0.0010** |
| **B²** | **224.72** | **224.72** | **25.49** | **< 0.0001** |
| **C²** | **71.17** | **71.17** | **8.07** | **0.0101** |
| **D²** | **80.32** | **80.32** | **9.11** | **0.0068** |
| **E²** | **248.64** | **248.64** | **28.20** | **< 0.0001** |
